# Genotype Mutations in Palestinian Children with Familial Mediterranean Fever: Clinical Profile, and Response to Colchicine Treatment: A Retrospective Cohort Study

**DOI:** 10.31138/mjr.20230912.stm

**Published:** 2023-09-12

**Authors:** Oadi N. Shrateh, Mariam Thalji, Afnan W.M. Jobran, Aml M. Brakat, Abdelrahman M. Attia, Fawzy M. Abunejma

**Affiliations:** 1 Faculty of Medicine, Al-Quds University, Jerusalem, Palestine,; 2 Faculty of Medicine, Zagazig University, Ash Sharqia Governorate, Egypt,; 3 Faculty of Medicine, Cairo University, Cairo, Egypt,; 4 Department of Paediatric Rheumatology, Palestinian Red Crescent Society Hospitals, Hebron, Palestine,; 5 Department of Paediatric Rheumatology, Al Ahli Hospital, Hebron, Palestine,; 6 Paediatric Department, Faculty of Medicine, Hebron University, Palestine

**Keywords:** Familial Mediterranean fever, Palestinian, genetics, clinical presentation, colchicine

## Abstract

**Background:**

Familial Mediterranean fever is a hereditary autoinflammatory disease affecting mainly Arabs, Turks, Armenians, and Jews with genotype-phenotype heterogeneity, presenting as recurrent episodes of fever along with polyserositis and rash. To date, more than 370 mutations in the *MEFV* gene have been recognized to cause the disease.

**Methods:**

We conducted a retrospective cohort study involving 124 patients in Hebron, Palestine, diagnosed with FMF at the Al-Ahli, and Palestinian Red Crescent Society (PRCS) Hospitals.

**Results:**

The median age of diagnosis was five years, presenting as abdominal pain (76.6%), fever (67.7%), joint pain and arthritis. Regarding *MEFV* gene mutations, we had 62 patients (50%) with heterozygous genotypes, 40 patients (32.3%) with homozygous phenotypes, 21 patients (16.9%) with compound heterozygous genotypes, and one was a missing state. Regarding variant frequencies, M694V was the most common one (43.4%), followed by E148Q (15.6%), V726A (5.7%), A744S (4.1%), and R202Q (4.1%). Positive family history was detected in 59 patients (54.6%), and there was no significant difference in zygosity regarding characteristics, consanguinity, and family history.

**Conclusions:**

We affirm in this study of 124 children with FMF, abdominal pain, followed by fever, joint pain and arthritis were the main manifestations. Further, M694V, E148Q, V726A, A744S, and R202Q were the most frequent mutations, and carrying the M649V mutations is associated with a predisposition to other comorbidities. We believe that this study gives a pervasive overview of FMF in Palestinian patients. Looking forward, future studies on a larger number of patients could precisely highlight the genotype-phenotype association among FMF patients.

## INTRODUCTION

Familial Mediterranean fever (FMF) is considered the most common inherited monogenic autoinflammatory periodic fever condition. FMF is additionally known as ‘periodic peritonitis’. Although FMF can affect any individual from different areas in the world, it frequently involves those originating from the East Mediterranean region and its surroundings including Arabs, Armenians, Turks, and Jews.^1–2^ FMF usually follows an autosomal recessive inheritance pattern which means that both alleles (homozygosity) should be mutated. Nevertheless, many patients have been diagnosed with FMF while having only one genetic mutation (heterozygosity) with significant response to the treatment. _3_ This genetic abnormality results in gaining of a new function in the *MEFV* gene.^4^

FMF is characterized by recurrent attacks of fever, polyserositis, arthritis, and rash, while Amyloidosis remains the most dreaded complication, especially if complicated by renal impairment.^5–6^ Episodic complaints begin before the age of 20, with each episode lasting from several hours to a few days. Headache is an accompanying complaint besides fever.^7^

The most recent criteria were published in 2020 entitled ‘Eurofever/PRINTO Classification Criteria’, which combines clinical manifestation and genetic finding together, making it more sensitive (96%) than Tel Hashomer (88.4%) and Yalcinkaya-Ozen criteria (93.4%), but less specific. According to Eurofever/PRINTO Classification Criteria, diagnosis of FMF can be established by the presence of confirmatory *MEFV* genotype and at least one of the following: (1) duration of episodes 1–3 days, (2) Chest pain, (3) Arthritis, (4) Abdominal pain or presence of non-confirmatory *MEFV* genotype and at least two of the following: (1) Duration of episodes 1–3 days, (2) Chest pain, (3) Arthritis, (4) Abdominal pain.^8^

Genetic and molecular analysis plays a fundamental role in establishing the diagnosis of clinically suspected FMF patients. The *MEFV* gene is located on the short arm of chromosome 16 and is considered the responsible gene in FMF.^9^ Approximately 370 variants of the *MEFV* gene have been discovered. Among these sequence variants, mutations in the M694I, M694V, E148Q, V726A, and M680I genes are responsible for the vast majority of FMF cases. 10% of patients diagnosed with FMF based on their clinical manifestations had been found to have no *MEFV* gene mutation.^10–13^

Since the 1970s, Colchicine has been considered the treatment of choice for FMF.^14^ The main goal of colchicine administration is to decrease and prevent the attacks frequency, making the patient symptoms-free between the episodes, and to prevent and halt amyloidosis development and progression.^15^ The severity of symptoms and treatment response are strongly correlated to the genetic variant, with the homozygous M694V mutation tend to have very severe disease and more colchicine resistance.^16^

This study aims to detect the genetic background of FMF, determine the most frequent *MEFV* mutations among Palestinians, and evaluate the clinical profile and the response to colchicine treatment with different genetic variants.

## MATERIALS AND METHODS

The current study is a double-centre, retrospective cohort survey that included 124 children and adolescents of both sexes (≤18 years) with the ascertained diagnosis of FMF according to their clinical manifestations and molecular basis, throughout their routine follow-up visits at the Rheumatology Outpatient Clinics of the paediatric department, Al-Ahli and Palestinian Red Crescent Society (PRCS) hospitals, Hebron, Palestine.

The study was carried out during the last two weeks of May 2022. This study aims to investigate the genetic background of FMF among our included patients and identify the most frequent *MEFV* genetic mutations, to study how different genetic mutations could affect the phenotypic expression of the disease, to detect the most frequent presenting manifestation, and to evaluate patient’s response to colchicine treatment with different genetic variants.

Diagnosis of FMF was established referring to the most recent criteria published in 2020 entitled ‘Eurofever/PRINTO Classification Criteria’ which combines clinical manifestation and genetic findings together. Patients who did not meet the criteria were excluded from the study.

All patients had a comprehensive personal and family history, physical and laboratory assessment, and after obtaining the approval to conduct this study, authors electronically reviewed and validated the data from the medical records. In case of incomplete or missing data, patients were reevaluated. The outcomes of *MEFV* molecular analysis were documented.

Subsequently, our patients were divided into either having homozygous (both alleles of the gene are affected), heterozygous (only one allele of the gene is affected) or compound heterozygous (mutated alleles on different genes). Genetic analysis was performed, to recognize the 11 most prevalent *MEFV* mutations; the M694V, M694I, E148Q, R202Q, M680I, R329H, V726A, P369S, SCN9A, A744S and A408G.

DNA was extracted from blood using QIAamp DNA mini kit followed by FMF targeted variants detection using FMF Multiplex real time PCR kit (Cat. No: 11R-20-13) from SNP Biotechnology.

### Data collection process

Medical records of patients were collected electronically from the hospital system, while some patients were face-to-face interviewed and re-evaluated.

### Statistical analysis

We evaluated the normality of the distribution of quantitative variables using the Shapiro-Wilks test. Data were statistically described in terms of median and interquartile range for quantitative variables with nonnormal distribution, and frequency and percentage for qualitative variables. In the absence of normal distribution, non-parametric tests used for the Comparison of numerical variables between the study groups were done using the Kruskal Wallis test followed by post hoc pairwise comparisons between groups using the Bonferroni adjustment method after significant effects have been found for comparing categorical data, the Chi-square (χ^2^) test was performed. A P-value less than 0.05 was considered statistically significant. We did all statistical calculations using R language programming software version 4.1.2 and Microsoft Excel 365.

## RESULTS

The omitted data has been presented in **Table 1** to elucidate any discrepancies or incongruities with the data presented in other tables. We identified 124 cases with familial Mediterranean fever and according to gene mutations and allele status, we classified our patients into three groups: the first group (homozygous group) included 40 patients of whom 16 (40%) were males and 24 (60%) were females, second group (heterozygous group) included 62 patients of whom 27 (43.5%) were males and 35 (56.5%) were females and third group (Compound Heterozygous) included 21 patients of whom 12 (60%) were males and 8 (40%) were females. The median age [IQR] of our population and median age at diagnosis of all study populations were 9.00 [6.00, 13.00], and 5.00 [3.00, 8.00] years respectively.

**Table 1:** Missing data.

**Variable**	**Overall (n=124)**	**Missing data**
**Age (years) (Median [IQR])**	9.00 [6.00, 13.00]	3
**Gender n (%)**		
**Female**	67 (54.5)	1
**Male**	56 (45.5)	
**Consanguinity n (%)**		
1**^st^ degree**	7 (6.3)	13
2**^nd^ degree**	5 (4.5)	
**None**	99 (8.2)	
**Family History of FMF n (%)**		
**Negative**	49 (45.4)	16
**Positive**	59 (54.6)	
**Age of presentation, (Median [IQR])**	5.00 [3.00, 8.00]	1
**Regimen mg/day (Median [IQR])**	1.00 [0.50, 1.00]	29

Comparing characteristics, consanguinity, and family history between different genetic zygosity, none of the parameters was statistically significant. Positive family history was reported in a higher percentage among Heterozygous 30 (56.6%), which was not significant. Most of our patients received colchicine therapy (n= 92, 74%); with a median dose of 1.00 [0.50, 1.00] mg/day, and 64 (69.6%) of our patients reported improvement. Homozygous and compound heterozygous patients were more likely to receive a higher dose of colchicine than heterozygous patients (median dose 1 vs. 0.88 mg/day) (**Table 2**).

**Table 2:** Demographic data showing differences between homozygotes, heterozygotes, and compound heterozygotes.

**Variable**	**Homozygous**	**Compound Heterozygous (n=21)**	**Heterozygous (n=62)**	**p-value**

	**(n=40)**			

**Age (years) (Median [IQR])**	9.75 [6.75, 13.00]	8.00 [6.75, 10.00]	8.50 [6.00, 13.00]	0.774

**Gender n (%)**				
**Female**	24 (60.0)	8 (40.0)	35 (56.5)	0.321
**Male**	16 (40.0)	12 (60.0)	27 (43.5)	

**Consanguinity n (%)**				
1**^st^ degree**	2 (5.7)	2 (11.1)	3 (5.3)	
2**^nd^ degree**	3 (8.6)	0 (0.0)	2 (3.5)	0.55
**none**	30 (85.7)	16 (88.9)	52 (91.2)	

**Family History of FMF n (%)**				
**Negative**	18 (48.6)	8 (47.1)	23 (43.4)	0.88
**Positive**	19 (51.4)	9 (52.9)	30 (56.6)	

**Age of presentation (Median [IQR])**	5.00 [2.00, 7.00]	5.00 [3.00, 9.00]	5.00 [4.00, 8.00]	0.399

**Regimen mg/day (Median [IQR])**	1.00 [1.00, 1.00]	1.00 [1.00, 1.00]	0.88 [0.50, 1.00]	0.01*

* P-value less than 0.05 is considered statistically significant; post-hoc testing using Bonferroni adjustment showed that the significance was between heterozygous and homozygous only (p=0.022). IQR: interquartile range; n: number of patients.

**Figure 1.**
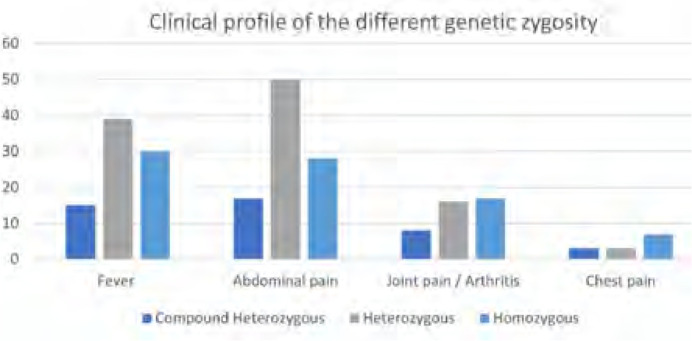
Clinical features of all patients of the different genetic zygosity.

Regarding the clinical features, abdominal pain was the most common presenting feature in 95 (76.6%) cases, followed by fever in 84 (67.7%). Joint pain and arthritis were common in both heterozygotes and the homozygotes groups and were documented in 16 (25.8%) and 17 (42.5%) patients, respectively (**Figure 1**). Regarding the consanguinity of the study population, 7 (6.3%) had consanguineous parents; 3 (5.3%) were heterozygous; 2 (5.7%) were homozygous, and 2 (11.1%) were compound heterozygous. We recorded a positive family history of FMF in 59 patients (54.6 %), of whom 30 (56.6%) were heterozygous and 19 (51.4%) were homozygous.

The most common seven mutations in our patients were (M694V, E148Q, V726A, A744S, R202Q, M694I, M694V + V726A, and M694V + R202Q). M694V was the most prevalent variant in the overall cohort 53 (43.4%) The M694V genotype distribution was homozygous in 32 (82.1%) and heterozygous in 21 (33.9%). The mutation E148Q was identified in 19 cases (15.6%), and all of these instances were found to be in a heterozygous state (30.6%) (**Table 3**).

**Table 3.** Gene mutations and Alleles analysis in FMF patients.

		**Total N (%)**	**Compound Heterozygous**	**Heterozygous**	**Homozygous**	**p value**
**Mutated gene (%)**	A744S	5(4.1)	0 (0.0)	5 (8.1)	0 (0.0)	
	E148Q	19 (15.6)	0 (0.0)	19 (30.6)	0 (0.0)	
	M694I	4 (3.3)	0 (0.0)	3 (4.8)	1 (2.6)	<0.001*
	M694V	53 (43.4)	0 (0.0)	21 (33.9)	32 (82.1)	
	M694V + R202Q	3 (2.5)	3 (14.3)	0 (0.0)	0 (0.0)	
	M694V + V726A	4 (3.3)	4 (19.0)	0 (0.0)	0 (0.0)	
	R202Q	5 (4.1)	0 (0.0)	1 (1.6)	4 (10.3)	
	V726A	7 (5.7)	0 (0.0)	7 (11.3)	0 (0.0)	

* P-values less than 0.05 are considered statistically significant.

FMF was associated with other disorders such as: juvenile idiopathic arthritis (JIA) in 3 patients having the M694V mutation; severe form of Henoch-Schoenlein purpura (HSP) was found in one patient having the M694V mutation; osteomyelitis was found in one patient having the V726A and E148Q mutations; Crohn’s disease was found in 3 patients having the M694V mutation; one patient had JIA and ankylosing spondylitis with the M694V mutation, and one patient had hypothyroidism with the V726A mutation. 32 (26.9%) of our cohort had microcytic anaemia, and most of them were either homozygous or heterozygous.

Overall, 39 (69.6%) reported improvement during the follow-up (Repetitive sentence); 8 (80%) of them were Compound Heterozygous; 17 (68%) were heterozygotes, and 14 (66.7%) were homozygotes. 17 (63%) of M694V patients reported improvement and 7(25.9%) had worsened clinical presentation, while Five patients with E148Q mutation reported improvement in their symptoms 5 (71.4%). Patients with M694V and R202Q mutations were more likely to present at a younger age than those with other mutations (**Table 4**).

**Table 4.** Demographic and clinical data, and response to colchicine in the most frequent mutations detected.

	**A744S (n=5)**	**E148Q (n=19)**	**M694I (n=4)**	**M694V (n=53)**	**M694V + R202Q (n=3)**	**M694V + V726A (n=4)**	**R202Q (n=5)**	**V726A (n=7)**	**p-value**

**Age (years) (median [IQR])**	8.00 [5.00, 8.50]	12.00 [9.25, 14.00]	10.25 [9.50, 11.62]	9.00 [6.00, 13.00]	7.00 [6.50, 8.50]	10.00 [7.50, 11.00]	8.00 [7.00, 10.00]	7.00 [5.50, 10.25]	0.784

**Gender n (%)**									0.693
**Female**	2 (40.0)	10 (52.6)	3 (75.0)	31 (58.5)	1 (33.3)	1 (25.0)	4 (80.0)	4 (57.1)	
**Male**	3 (60.0)	9 (47.4)	1 (25.0)	22 (41.5)	2 (66.7)	3 (75.0)	1 (20.0)	3 (42.9)	

**Fever (%)**	3 (60.0)	10 (52.6)	1 (25.0)	35 (66.0)	2 (66.7)	3 (75.0)	5 (100.0)	7 (100.0)	0.422

**Abdominal pain (%)**	5 (100.0)	15 (78.9)	3 (75.0)	37 (69.8)	2 (66.7)	4 (100.0)	5 (100.0)	7 (100.0)	0.308

**Joint pain and/or arthritis (%)**	1 (20.0)	4 (21.1)	1 (25.0)	21 (39.6)	2 (66.7)	1 (25.0)	1 (20.0)	2 (28.6)	0.614

**Chest pain (%)**	0 (0.0)	1 (5.3)	0 (0.0)	7 (13.2)	1 (33.3)	0 (0.0)	0 (0.0)	1 (14.3)	0.224

**Regimen mg/day (median [IQR])**	0.75 [0.50, 1.00]	1.00 [0.62, 1.00]	0.75 [0.38, 1.00]	1.00 [0.50, 1.00]	1.00 [1.00, 1.00]	1.00 [0.88, 1.00]	1.00 [0.88, 1.00]	0.50 [0.50, 0.50]	0.422

**Follow up (%)**									
**Improving**	0 (0.0)	5 (71.4)	2 (100.0)	17 (63.0)	1 (50.0)	2 (100.0)	3 (100.0)	1 (50.0)	
**Unchanged**	0 (0.0)	0 (0.0)	0 (0.0)	3 (11.1)	0 (0.0)	0 (0.0)	0 (0.0)	0 (0.0)	0.989
**Worsening**	1 (100.0)	2 (28.6)	0 (0.0)	7 (25.9)	1 (50.0)	0 (0.0)	0 (0.0)	1 (50.0)	

**Consanguinity (%)**									
1**^st^ degree**	1 (20.0)	1 (5.6)	0 (0.0)	1 (2.2)	0 (0.0)	0 (0.0)	1 (20.0)	1 (16.7)	
2**^nd^ degree**	0 (0.0)	1 (5.6)	0 (0.0)	2 (4.3)	0 (0.0)	0 (0.0)	0 (0.0)	0 (0.0)	0.015*
**None**	4 (80.0)	16 (88.9)	4 (100.0)	43 (93.5)	3 (100.0)	3 (100.0)	4 (80.0)	5 (83.3)	

**Family history of**									
**FMF (%)**									
**Negative**	2 (40.0)	11 (64.7)	1 (25.0)	16 (34.8)	2 (66.7)	1 (33.3)	4 (80.0)	4 (66.7)	
**Positive**	3 (60.0)	6 (35.3)	3 (75.0)	30 (65.2)	1 (33.3)	2 (66.7)	1 (20.0)	2 (33.3)	0.329

**Age of presentation (years) (median [IQR])**	7.50 [4.00, 8.00]	7.00 [4.00, 9.00]	8.00 [6.25, 10.00]	5.00 [2.50, 6.50]	3.00 [2.95, 4.50]	3.50 [2.75, 5.25]	4.00 [4.00, 7.00]	4.00 [3.00, 7.70]	0.792

* P-values less than 0.05 are considered statistically significant. IQR: interquartile range; n: number of patients.

We employed Direct DNA sequencing to examine the exons of all individuals diagnosed with FMF, aiming to identify any mutations potentially responsible for the disease. Among the FMF patients, 80 exhibited an exon 10 mutation, primarily involving four variants: M694V, M694I, V726A, and M680I, which collectively accounted for the majority of cases. In addition, we detected 27 cases with mutations outside of exon 10. Notably, 15 individuals displayed a combination of mutations, encompassing both exon-10 and non-exon-10 mutations. According to laboratory findings, elevated inflammatory markers were noticed more apparently in those with exon-10 mutations than others as outlined in **Table 5.**

**Table 5.** Phenotypic comparison between FMF patients carrying exon-10 mutations and those carrying non-exon-10 mutations.

		**Exon-10 (n= 80)**	**Mixed (n=15)**	**Non-exon-10 (n=27)**	**p**
**Physical examination**					
**Fever (%)**		55 (68.8)	10 (66.7)	18 (66.7)	0.973
**Abdominal pain (%)**		62 (77.5)	10 (66.7)	22 (81.5)	0.542
**Joint pain and/or arthritis (%)**		30 (37.5)	5 (33.3)	5 (18.5)	0.192
**Chest pain (%)**		10 (12.5)	2 (13.3)	1 (3.7)	0.413
**Normal (%)**		58 (72.5)	7 (50.0)	16 (61.5)	0.193
**Swollen ankle (%)**		3 (3.8)	1 (7.1)	0 (0.0)	0.456
**Abdominal distension and tenderness (%)**		2 (2.5)	2 (14.3)	3 (11.5)	0.083
**Tender joint + limited movement (%)**		7 (8.8)	1 (7.1)	1 (3.8)	0.711
**Laboratory investigation**					
**Insignificant (%)**		37 (46.2)	3 (21.4)	9 (37.5)	0.199
**High CRP (%)**		30 (37.5)	4 (28.6)	7 (29.2)	0.66
**High ESR (%)**		32 (40.0)	6 (42.9)	9 (37.5)	0.947
**Microcytic anemia (%)**		24 (30.0)	4 (28.6)	3 (12.5)	0.227
	Compound	9 (11.2)	12 (80.0)	0 (0.0)	<0.001
**Genetic zygosity (%)**	Heterozygous				
	Heterozygous	37 (46.2)	3 (20.0)	22 (81.5)	
	Homozygous	34 (42.5)	0 (0.0)	5 (18.5)	

**Figure 2.**
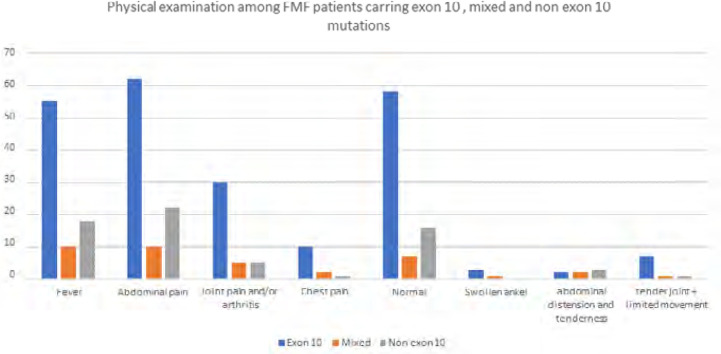
Differences in the physical findings among FMF patients carrying exon-10, mixed, and non-exon-10 mutations.

**Figure 3.**
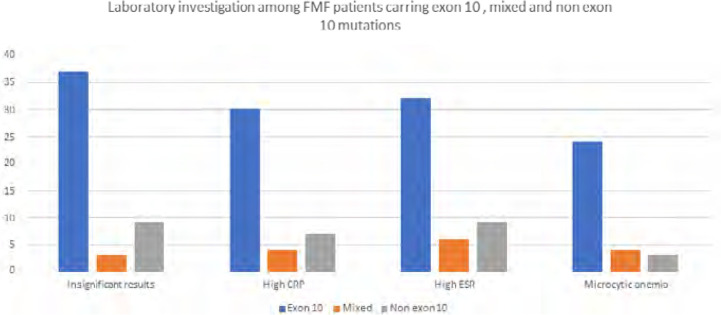
Differences in the laboratory findings among FMF patients carrying exon-10, mixed, and non-exon-10 mutations.

When reviewing the physical examination records, instances of abdominal pain were most frequently reported in the exon 10 mutant group (62 cases, 77.5%), followed by the non-exon mutation group (22 cases, 81.5%), and the mixed group (10 cases, 66.7%). Similarly, concerning fever, the exon 10 mutation group (55 cases, 68.8%), exhibited a higher frequency of reported occurrences compared to the non-exon 10 group (18 cases, 66.7%), as illustrated in **Figures 2** and **3.**

## DISCUSSION

FMF is the most common hereditary autoinflammatory disease, affecting Arabs, Turks, Armenians, and non-Ashkenazi Jews from the East Mediterranean region and surrounding areas.^17–18^ Although this disease affects Arab societies, there is no sufficient data regarding the prevalence of FMF in Arab countries, including Palestine, where the study was done.^19^ In this study, we aim to identify the most frequent *MEFV* mutations as well as to evaluate the clinical profile and response to colchicine treatment in patients with genetic variance.

Our analysis involved 124 paediatric patients, 67 female (54.5%) and 56 male (45.5%), diagnosed with FMF and positive *MEFV* mutations. In agreement with Talaat et al. and Duşunsel et al.^20–21^ female-to-male ratio was found to be high (1.2:1) compared to other studies that reported a male predominance with a male-to-female ratio of 1.2: 1 in Turkish populations,^22–23^ or equal prevalence between Arab males and females.^24^

It is of interest that the median age of diagnosis in our study was five years old, which is consistent with other studies that diagnosed FMF before the age of 10.^25^ Meanwhile, studies conducted in Egypt and Turkey have reported 7 and 10 years old as the median age of diagnosis.^20–21,26^ Surprisingly, two FMF patients in this study were diagnosed before the age of one year, which was challenging due to the lack of verbal milestones expressing their complaints on top of the ambiguous presentation. Hence, diagnosing FMF in younger paediatric age groups, particularly in endemic regions, is crucial to decrease late comorbidities.^27^

Various genotypes are reflected in FMF patients with different ethnicities. To date, more than 370 *MEFV* genes are involved in the development of the disease.^13^ In the Middle East, M694I, M694V, V726A, E148Q, and M680I gene variants are responsible for most FMF cases, and the allele frequency for these genes is highly variable even between the same country.^12^ Our study has revealed M694V (43.4%) as the most frequent mutation, followed by E148Q (15.6%), V726A (5.7%), A744S (4.1%), R202Q (4.1%), M694I (3.3%), and M694V + V726A (3.3%). This somehow corresponds to a survey conducted on a Lebanese population with the same mutational profile but with different frequencies, where M694V (20.6%) was found to be the most common mutation, followed by E148Q (17.9%) and V726A (16.0%). R202Q (12.6%), M694I (11.8%), and A744S (10.7%).^28^ Similarly, previous reports in various Arab countries, including Syria, Jordan, Morocco, and Tunisia,^29–33^ found that the M694V variant was the predominant genotype among FMF patients.

Compared with a previous Palestinian study,^34^ we discovered that M694V was the leading mutation, followed by V726A, M694I, E148Q, and M680I mutations. However, there were some discrepancies in frequencies; for example, the E148Q mutation, which was the second most prevalent variant in our study (15.6%), was found to be the fourth most prevalent variant in the Palestinian study (8.5%).^34^ Another notable point is that the R202Q genotype among Arabs was only documented in our study and another study conducted on Lebanese patients. Further, the same R202Q genotype was reported in a study done by Coşkun et al. and Gunesacar et al. for Turkish patients.^35–36^ In comparison, studies from Egypt revealed different results, with E148Q and M694I mutations being the most observed.^37–38^

A positive family history of FMF was documented in 59 patients (54.6 %), of whom 30 were heterozygous, and 19 were homozygous. These findings appear to be high when compared to studies performed by Jarjoure and Al Berrawi,^30^ and Talaat et al.^20^ who detected a positive family history in 32 (31 %) out of their 103 Syrian patients, and 21 (22.11%) out of their 95 Egyptian patients, respectively.

While parental consanguinity was evident in 6.3 % of our study population, this had no effect on genetic zygosity. However, Jarjoure and Al Berrawi,^30^ and Talaat et al.^20^ reported a higher consanguinity rate in their population as (39.8 %), and 37.89%, respectively.

Several studies found an association between FMF and other inflammatory diseases, most notably juvenile idiopathic arthritis (JIA), Henoch–Schönlein purpura (HSP), and inflammatory bowel diseases (IBD).^39–41^ Approximately 7.25% of FMF patients in this study had other concomitant diseases, corresponding to a study done by Ayaz et al.^42^ This could be attributed to the persistence of a subclinical inflammatory state between the attacks,^22,40–44^ promoting generalized inflammation and triggering the onset of other auto-inflammatory diseases. Our results indicated that FMF was associated with JIA in 3 patients (2.41%), Crohn’s disease in 3 patients (2.41%), a severe form of HSP in one patient (0.8%), both JIA and ankylosing spondylitis in one patient (0.8%) and hypothyroidism in one patient (0.8%).

Carrying exon 10 mutations, such as the M694V mutation, appears to predispose FMF patients to the development of certain inflammatory diseases, which was consistent with our findings. According to Yıldız et al., 18.9% of their Turkish FMF patients have a co-existing condition, the most prevalent of which were uveitis (n = 12, 1.7%), asthma (n=29, 4.2%), JIA (n=42, 6.1%), IgA vasculitis (n = 20, 2.9%), and inflammatory bowel disease (n=10, 1.4%). However, only IBD and asthma patients had the *MEFV* gene mutation.46 In contrast to our results, a study among 95 Egyptian patients mentioned that 13.68% of their patients had other inflammatory conditions such as JIA (n=4, 4.21%), HSP in (n=3, 3.16%), SLE (n=3,3.16%), and IBD (n=2, 2.11%), while the most frequent correlated mutation was M680I followed by E148Q, V726A and M694V mutations.^20^

About 26.9% of our patients had microcytic anaemia, while anaemia in FMF patients can be functional, due to chronic inflammation, absolute iron deficiency (AID), or a combination of both, differentiating between them is essential, as iron therapy alone is adequate to improve anaemia in FMF patients with AID, but not in the other types.^47^ Further, Celkan et al.28 mentioned that Colchicine therapy has a role in the treatment of anaemia via suppression of the active inflammatory process.

Regarding the clinical presentation of FMF, abdominal pain is the known presenting complaint among Arab patients.^25^ These observations are in agreement with our study in 95 (76.6%) of cases, followed by fever in 84 (67.7%), which is similar to other studies conducted in Syria and Turkey.^22–23,30^ Joint pain and arthritis were a shared feature in both heterozygotes and homozygotes group, and were documented in 16 (25.8%), and 17 (42.5%) patients, respectively. However, fever was the most common complaint among 109 Egyptian patients (96.84%) in a study done by Talaat et al.^20^ followed by abdominal pain (94.74%), and arthritis (78.95%).

Our aim regarding treatment is to prevent recurrent attacks, reduce the inflammatory condition and minimise the risk of developing late complications. Since 1972, colchicine has been the cornerstone of treatment due to its effectiveness in reducing the number and the severity of attacks as well as preventing complications such as amyloidosis.^15,22,49^ The European Alliance of Associations for Rheumatology (EULAR) guidelines recommend a dose of: 0.5 mg/day for children under 5 years of age, 0.5–1.0 mg/day in children 5–10 years: and 1.0–1.5 mg/day for children >10 years and adults.^50^ Most of our patients received colchicine therapy (n= 92, 74%); with a median dose of 1.00 [0.50, 1.00] mg/day, and 64 (69.6%) of our patients reported improvement. Homozygous and compound heterozygous patients were more likely to receive a higher dose of colchicine than heterozygous patients (median dose 1 vs. 0.88 mg/day). Homozygotes and Compound Heterozygous have found required a higher dose to control attacks 1.00 [1.00, 1.00] mg/day, as compared to heterozygotes 0.88 [0.50, 1.00] mg/day (p-value= 0.01). Overall, 39 (69.6%) reported improvement during the follow-up; 8 (80%) of them were Compound Heterozygous; 17 (68%) were heterozygotes, and 14 (66.7%) were homozygotes. Similarly, an Egyptian study conducted among 70 patients revealed that the required dose for attacks control was significantly lower in heterozygotes than homozygotes, with a better response to colchicine in the heterozygous group.^51^

We recognise that there are some limitations to our study. First, the small sample size as we cannot find data consistent with ours in terms of detailed clinical features, diagnosis, and treatment at other hospitals. Second, the exclusion of many patients diagnosed with FMF due to incompliance with the therapeutic regimen and follow-up visits.

Eventually, prospective studies on larger scales are needed to screen for other unique and newly addressed variants as well as scrutinise those of ambiguous significance in order to obtain a more precise diagnosis of FMF and thus provide patients with individualised treatment and enhanced disease management outcomes.

## CONCLUSION

M694V, E148Q, V726A, A744S, and R202Q were the most common mutations in our patients. Specifically, M694V was the most prevalent variant in the overall cohort. Approximately half of our patients were found to a have heterozygote state. Further, Abdominal pain was the presenting feature among our patients, while Fever, joint pain, and arthritis were also observed as clinical manifestations. FMF syndrome may be associated with other autoimmune diseases as a result of the persisting inflammatory state. Regardless of our current FMF knowledge, we recommend further investigation of a larger number of patients in order to further clarify the genotype-phenotype correlation among Palestinian FMF paediatric patients.

## Data Availability

The dataset used and analysed during the current study is available from the corresponding author on reasonable request.

## Abbreviations:

AID: Absolute Iron Deficiency
FMF: Familial Mediterranean Fever
HSP: Henoch–Schönlein Purpura
IBD: Inflammatory Bowel Diseases
MEFV: Mediterranean Fever
PCR: Polymerase Chain Reaction
PRCS: Palestinian red crescent
SAA: Serum Amyloid A
SNP: Single Nucleotide Polymorphisms
